# Epigenetic Control of *Salmonella enterica* O-Antigen Chain Length: A Tradeoff between Virulence and Bacteriophage Resistance

**DOI:** 10.1371/journal.pgen.1005667

**Published:** 2015-11-19

**Authors:** Ignacio Cota, María Antonia Sánchez-Romero, Sara B. Hernández, M. Graciela Pucciarelli, Francisco García-del Portillo, Josep Casadesús

**Affiliations:** 1 Departamento de Genética, Facultad de Biología, Universidad de Sevilla, Sevilla, Spain; 2 Centro Nacional de Biotecnología (CNB-CSIC), Madrid, Spain; 3 Departamento de Biología Molecular, Universidad Autónoma de Madrid, Centro de Biología Molecular Severo Ochoa (CBMSO-CSIC), Madrid, Spain; Seoul National University, REPUBLIC OF KOREA

## Abstract

The *Salmonella enterica opvAB* operon is a horizontally-acquired locus that undergoes phase variation under Dam methylation control. The OpvA and OpvB proteins form intertwining ribbons in the inner membrane. Synthesis of OpvA and OpvB alters lipopolysaccharide O-antigen chain length and confers resistance to bacteriophages 9NA (Siphoviridae), Det7 (Myoviridae), and P22 (Podoviridae). These phages use the O-antigen as receptor. Because *opvAB* undergoes phase variation, *S*. *enterica* cultures contain subpopulations of *opvAB* ^OFF^ and *opvAB* ^ON^ cells. In the presence of a bacteriophage that uses the O-antigen as receptor, the *opvAB* ^OFF^ subpopulation is killed and the *opvAB* ^ON^ subpopulation is selected. Acquisition of phage resistance by phase variation of O-antigen chain length requires a payoff: *opvAB* expression reduces *Salmonella* virulence. However, phase variation permits resuscitation of the *opvAB* ^OFF^ subpopulation as soon as phage challenge ceases. Phenotypic heterogeneity generated by *opvAB* phase variation thus preadapts *Salmonella* to survive phage challenge with a fitness cost that is transient only.

## Author Summary

A tradeoff can increase the adaptive capacity of an organism at the expense of lowering the fitness conferred by specific traits. This study describes a tradeoff that confers bacteriophage resistance in *Salmonella enterica* at the expense of reducing its pathogenic capacity. Phase variation of the *opvAB* operon creates two subpopulations of bacterial cells, each with a distinct lipopolysaccharide structure. One subpopulation is large and virulent but sensitive to phages that use the lipopolysaccharide O-antigen as receptor, while the other is small and avirulent but phage resistant. In the presence of a phage that targets the O-antigen, only the avirulent subpopulation survives. However, phase variation permits resuscitation of the virulent *opvAB* ^OFF^ subpopulation as soon as phage challenge ceases. This transient tradeoff may illustrate the adaptive value of epigenetic mechanisms that generate bacterial subpopulations in a reversible manner.

## Introduction

The study of differentiation in bacterial species that undergo developmental programs has played a historic role in biology [1,2,3]. In addition, phenotypic differences between colonies [4] and within colonies [5,6] were described many years ago in bacterial species that do not undergo development. Despite their technical limitations, these early studies contributed to bring about the idea that phenotypic heterogeneity might be a common phenomenon in the bacterial world [7]. This view has been confirmed by single cell analysis technologies [8,9,10,11,12]. Furthermore, theoretical analysis has provided evidence that phenotypic heterogeneity can have adaptive value, especially in hostile or changing environments [13,14,15]. In certain cases, the adaptive value of subpopulation formation is illustrated by experimental evidence [16,17,18].

Formation of bacterial lineages is governed by diverse mechanisms, including programmed genetic rearrangement [19] and contraction or expansion of DNA repeats at genome regions [20,21]. In other cases, however, lineage formation is controlled by epigenetic mechanisms: certain cell-to-cell differences serve as physiological signals, and signal propagation by a feedback loop generates an inheritable phenotype [12,22]. Cell-to-cell differences can be a consequence of environmental inputs or result from the noise intrinsical to many cellular processes [10,12,15]. In turn, the feedback loops that propagate the initial state beyond can be relatively simple or involve complex mechanisms like the formation of inheritable DNA adenine methylation patterns in the genome [12,23,24]. Some feedback loops are stable enough to cause bistability, the bifurcation of a bacterial population into two distinct phenotypic states [22].

If a feedback loop is metastable, reversion of the epigenetic state will occur after a certain number of cell divisions. Reversible bistability is usually known as phase variation, and typically involves reversible switching of gene expression from OFF to ON or from low to high expression [25,26,27]. Examples of phase variation have been described mostly in bacterial pathogens, and subpopulation formation is frequently viewed as a strategy that may facilitate evasion of the immune system during infection of animals [25,26]. This view is supported by the observation that phase-variable loci often encode envelope components or proteins involved in modification of the bacterial envelope [25,26].

Some phase-variable envelope modifications controlled by DNA adenine methylation play roles in bacteriophage resistance. For instance, phase variation in the *gtrABC1* cluster protects *S*. *enterica* against the T5-like phage SPC35, probably by an indirect mechanism [28]. In *Haemophilus influenzae*, DNA adenine methylation controls phase-variable resistance to bacteriophage HP1c1 but the underlying mechanism remains hypothetical [29]. Phase variation can also contribute to phage resistance without alteration of the bacterial surface. For instance, certain genes encoding restriction-modification systems show phase variation [30,31].

In this study, we describe a phase variation system that confers resistance to bacteriophages that use the lipopolysaccharide (LPS) O-antigen as receptor. The genome of *Salmonella enterica* contains a horizontally-acquired locus, known as *opvAB* or *STM2209-STM2208* [32]. The *opvA* and *opvB* genes form a bicistronic operon [32] and encode inner membrane proteins [32]. OpvA is a small peptide of 34 amino acids, and OpvB is a larger protein of 221 amino acids with homology to the Wzz superfamily of regulators of LPS O-antigen chain length [32]. We show that expression of the *S*. *enterica opvAB* operon confers resistance to bacteriophages P22 (Podoviridae), 9NA (Siphoviridae), and Det7 (Myoviridae) by modification of the phage receptor, the LPS O-antigen. Because expression of *opvAB* is phase-variable, bacteriophage resistance occurs in the subpopulation of *opvAB* ^ON^ cells only. This subpopulation, which is extremely small, preadapts *Salmonella* to survive phage challenge albeit at the cost of reduced virulence. However, because the *opvAB* ^ON^ state is reversible, the virulence payoff is temporary, and a virulent bacterial population resuscitates as soon as phage challenge ceases.

## Results

### Localization of the OpvA and OpvB proteins in the *S*. *enterica* envelope

**Fig 1:**
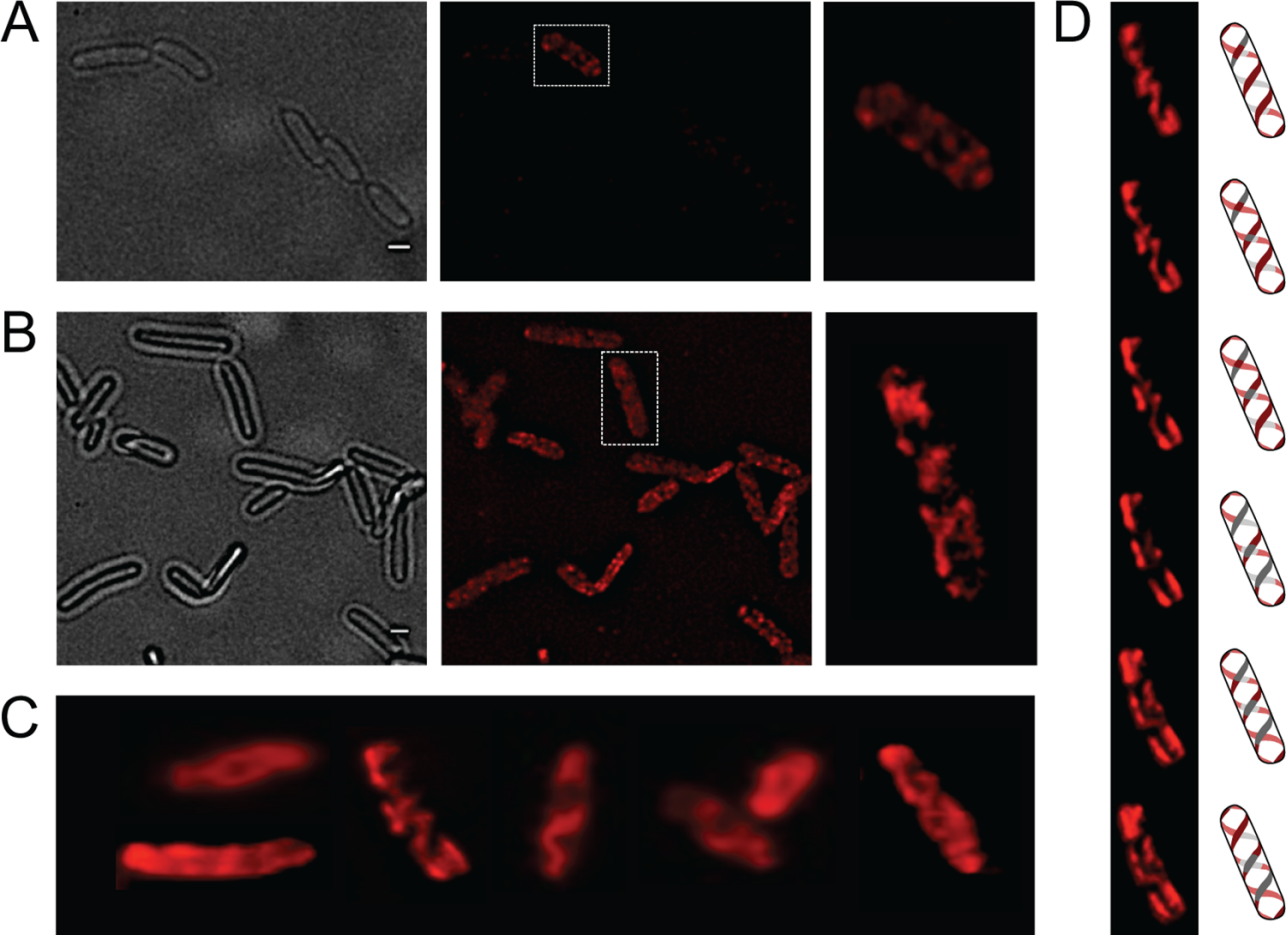
Analysis of OpvA and OpvB localization by fluorescence microscopy. **A.** Localization of OpvB-mCherry in a wild type background. **B**. Localization of OpvB-mCherry in an *opvAB* ^ON^ strain. In both panels, cells enclosed in boxes are shown with higher magnification on the right. **C.** Localization of plasmid-borne OpvA-mCherry. **D.** Z-stacks of a single cell from panel C accompanied by an idealized representation of the ribbon-like protein distribution. Scale bar: 1 μm.

OpvA and OpvB were previously shown to be inner membrane proteins involved in LPS synthesis [32]. Because the LPS is known to have a helical distribution in the cell envelope [33], the OpvA and OpvB subcellular localization was investigated. For this purpose, a chromosomal *opvB*::mCherry fusion was constructed downstream of the *opvB* gene (so that the strain remains OpvAB^+^). In a wild type background, expression of *opvB*::mCherr*y* was low in most cells (Fig 1A). However, rare cells with high levels of expression of *opvB*::mCherry were detected (Fig 1A), an observation consistent with the occurrence of phase variation skewed towards the OFF state [32]. Expression of *opvB*::mCherry was also monitored in an *opvAB*-constitutive (*opvAB* ^ON^) strain engineered by elimination of GATC sites upstream of the *opvAB* promoter [32]. In an *opvAB* ^ON^ background, all cells displayed high levels of fluorescence, similar to those of the rare fluorescent cells visualized in a wild type background (Fig 1B). In fluorescent cells, OpvB was seen forming helical intertwining ribbons in the inner membrane (Fig 1A and 1B).

The subcellular distribution of OpvA was examined using a plasmid-borne *opvA*::mCherry fusion. This experimental choice was based on the consideration that construction of an mCherry fusion in the upstream gene *opvA* would likely prevent *opvB* expression because of a polarity effect. In the strain carrying plasmid-borne *opvA*::mCherry, intense fluorescence was observed in all cells (Fig 1C), presumably because *opvA*::mCherry overexpression from the multicopy plasmid abolished phase variation. This construction was useful, however, to permit clear-cut observation of helical intertwining ribbons formed by OpvA (Fig 1D). The evidence that OpvA and OpvB may have a similar or identical distribution in the bacterial envelope is consistent with the physical interaction previously described between OpvA and OpvB [32].

### Roles of OpvA and OpvB in control of O-antigen chain length

**Fig 2:**
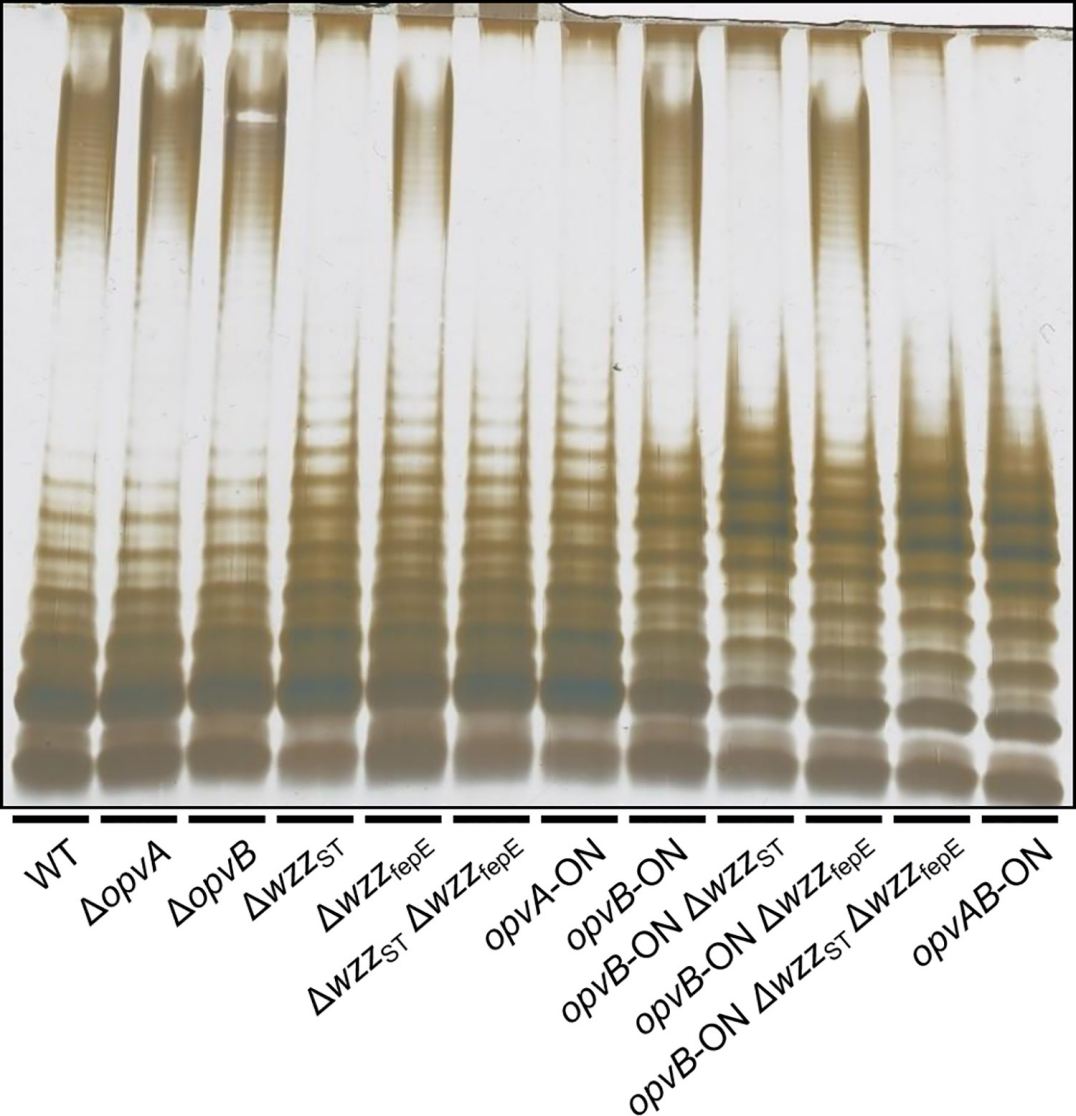
Analysis of the roles of OpvA and OpvB in the control of O-antigen chain length. LPS profiles of *S*. *enterica* strains carrying mutations in *opvAB* and O-antigen length regulator genes *wzz*_ST_ and *wzz*_fepE_, as observed by electrophoresis and silver staining.

Constitutive expression of *opvAB* leads to the production of a particular form of O-antigen in the *S*. *enterica* LPS, with a modal length of 3–8 repeat units [32]. A diagram of LPS structure is presented in S1 Fig, together with an electrophoretic separation of O-antigen chains and a diagram of the differences in LPS structure between *opvAB* ^OFF^ and *opvAB* ^ON^ pubpopulations. To investigate the role of individual OpvA and OpvB proteins in control of O-antigen chain length, non-polar mutations in *opvA* and *opvB* were constructed in the wild type and in an *opvAB* ^ON^ background. In the wild type, lack of either OpvA or OpvB did not alter the electrophoretic profile of LPS (Fig 2), an observation consistent with two known facts: the subpopulation of cells that express *opvAB* in wild type *Salmonella* is very small [32], and an OpvAB^−^mutant displays an LPS profile identical to that of the wild type [32]. In contrast, OpvA^−^ *opvB* ^ON^ and *opvA* ^ON^ OpvB^−^mutants showed differences with the parental *opvAB* ^ON^ strain and also with the wild type:

1. Absence of OpvB (*opvA* ^ON^) yielded a seemingly disorganized LPS with no clear modal length (Fig 2), reminiscent of the LPS produced in the absence of the modal length regulators Wzz_ST_ and Wzz_fepE_ [34,35,36].
2. Absence of OpvA (*opvB* ^ON^) yielded an LPS with the modal lengths typically conferred by Wzz_ST_ and Wzz_fepE_ [34] but also showed a preferred *opvAB* ^ON^-like modal length in the lower weight band region (Fig 2).

These observations suggested that the function of OpvA might be to prevent the formation of normal O-antigen so that OpvB could then impose its preferred modal length. To test this hypothesis, LPS structure was analyzed in an OpvA^*−*^ *opvB* ^ON^ background in the absence of either Wzz_ST_ or Wzz_fepE_. The results support the view that OpvB needs OpvA to prevent O-antigen formation by customary modal length regulators. In the absence of Wzz_ST_, OpvB alone was able to produce an O-antigen similar to that found in the *opvAB* ^ON^ strain (Fig 2). In contrast, lack of Wzz_fepE_ did not seem to facilitate OpvB function, suggesting that OpvB may mainly compete with Wzz_ST_. This preference may be related to the fact that both Wzz_ST_ and OpvB convey relatively short preferred modal lengths: 3–8 for OpvB [32] and 16–35 for Wzz_ST_ [36,37,38] compared with >100 for Wzz_fepE_ [34].

### Selection of *opvAB* ^ON^ *S*. *enterica* cells upon bacteriophage challenge

**Fig 3:**
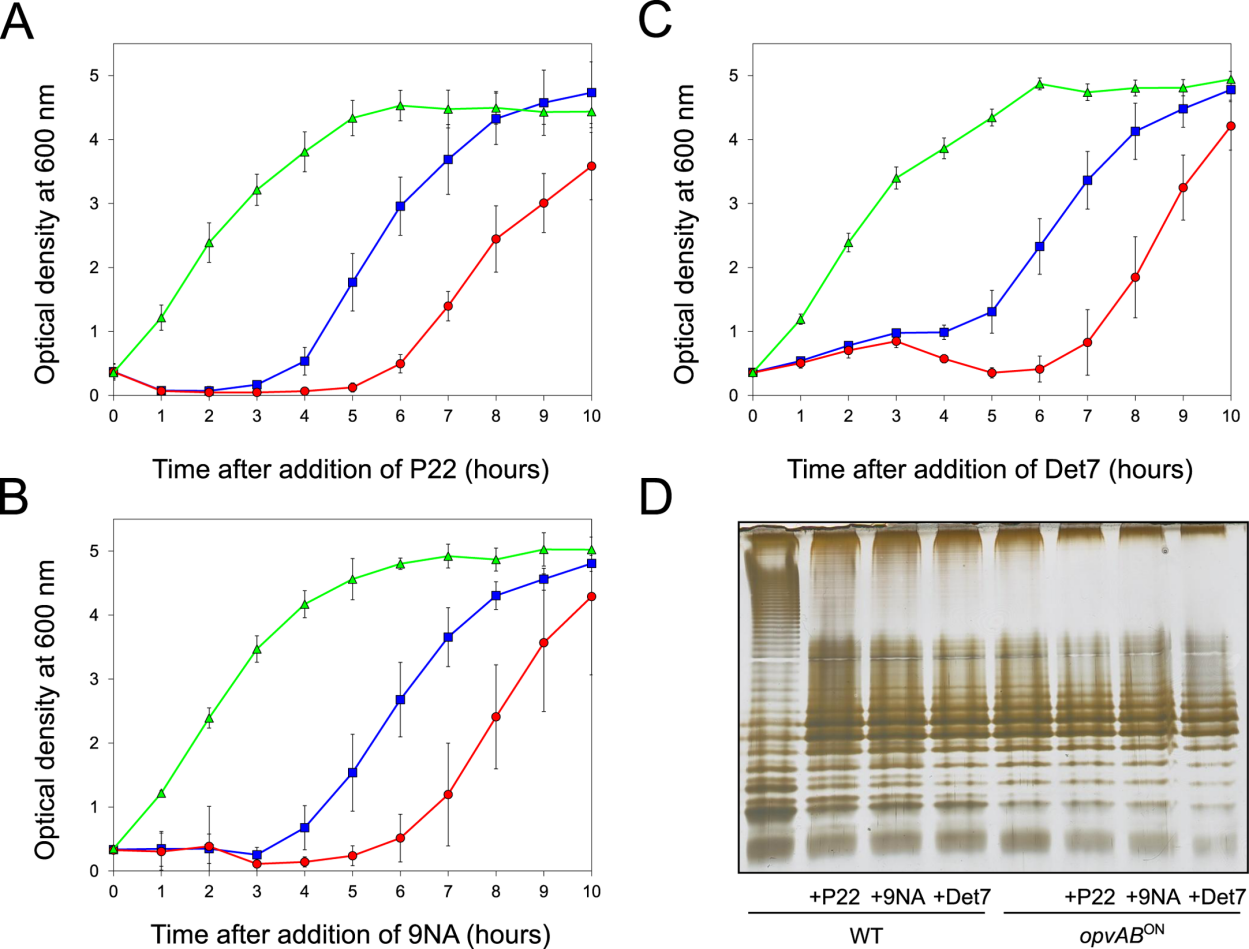
Effect of phage challenge on *S*. *enterica* growth and LPS structure. Growth of the wild type strain (blue squares), an *opvAB* ^ON^ strain (green triangles) and a Δ*opvAB* strain (red circles) in LB + P22 (**A**), LB + 9NA **(B),** and LB + Det7 **(C)**. Values are averages and standard deviations from ≥ 6 independent experiments. **D.** LPS structure of the wild type and *opvAB* ^ON^ strains after growth in LB, LB + P22, LB + 9NA, and LB + Det7.

The LPS O-antigen is a typical receptor for bacteriophages [39] and modification of the O-antigen can confer bacteriophage resistance [40]. On these grounds, we tested whether *opvAB* expression increased *Salmonella* resistance to the virulent phages 9NA [41,42] and Det7 [43,44]. We also tested the historic phage P22, using a virulent mutant to avoid lysogeny [45]. Three strains (wild type, *opvAB* ^ON^ and Δ*opvAB*) were challenged with 9NA, Det7, and P22, which belong to different bacteriophage families and use the O-antigen as receptor. The experiments shown in Fig 3 were carried out by inoculating an exponential culture of *S*. *enterica* with an aliquot of a phage suspension at a multiplicity of infection (MOI) >10, and monitoring bacterial growth afterwards. The results can be summarized as follows:

1. Growth of the *opvAB* ^ON^ strain was not affected by the presence of 9NA, Det7, or P22, suggesting that the strain was resistant to these bacteriophages (S2 Fig).
2. A culture of the wild type strain became clear 1–2 hours after P22 infection, suggesting that cell lysis had occurred. However, bacterial growth was observed around 4 hours after infection, and was interpreted as occurrence of P22 resistance. Infection with either 9NA or Det7 did not cause clearing but growth retardation, and growth resumed 4 hours after infection (Fig 3).
3. Cultures of the Δ*opvAB* strain infected with 9NA, Det7, or P22 became clear or almost clear. Growth was detected later, albeit with significant delay compared with the wild type. Because the wild type, the *opvAB* ^ON^ strain and the Δ*opvAB* strain show similar or identical growth rates in LB broth (S2 Fig), the explanation of this phenomenon is that growth of the Δ*opvAB* strain in the presence of 9NA, Det7, or P22 selects phage-resistant mutants (see below).

A tentative interpretation of these observations was that the wild type strain contained a subpopulation of *opvAB* ^ON^ cells that survived phage challenge. Because *opvAB* phase variation is skewed towards the OFF state [32], the small size of the *opvAB* ^ON^ subpopulation and the regular formation of phage-sensitive *opvAB* ^OFF^ cells caused growth retardation (albeit to different degrees depending on the phage). In contrast, the *opvAB* ^ON^ strain grew normally, an observation consistent with the occurrence of phage resistance in the entire bacterial population. This interpretation was supported by analysis of the LPS profiles of wild type and *opvAB* ^ON^ strains grown in the presence of P22, 9NA, and Det7 until stationary phase (OD_600_ ~4) (Fig 3). After phage challenge, the wild type contained an LPS different from the LPS found in LB (Fig 3D), and similar or identical to the LPS found in the *opvAB* ^ON^ strain (Fig 2; see also [32]). In contrast, the LPS from the *opvAB* ^ON^ strain did not change upon phage challenge (Fig 3D).

**Fig 4:**
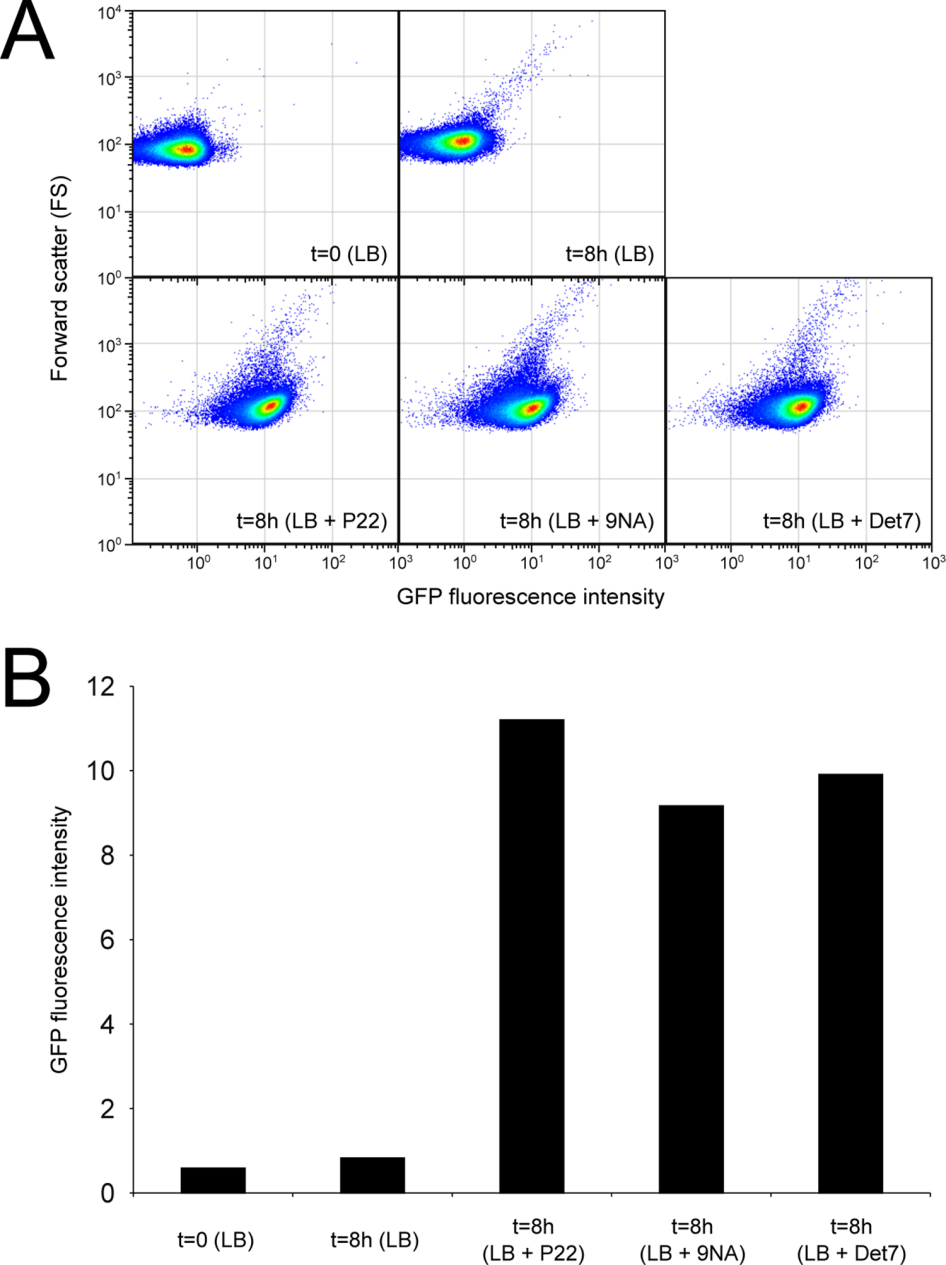
Flow cytometry analysis of *opvAB* ^OFF^ and *opvAB* ^ON^ subpopulations. **A.** GFP fluorescence distribution in an ATCC 14028 derivative carrying an *opvB*::*gfp* fusion before (t = 0) and after growth in LB, LB + P22, LB + 9NA, and LB + Det7 (t = 8h). Data are represented by a dot plot (forward scatter [cellular size] versus fluorescence intensity [*opvB*::*gfp* expression]). All data were collected for 100,000 events per sample. B. Medians of GFP fluorescence from the same experiments.

Confirmation that challenge of the wild type with P22, 9NA, and Det7 selected *opvAB* ^ON^ *S*. *enterica* cells was obtained by flow cytometry analysis (Fig 4). Expression of *opvAB* was monitored using a green fluorescent protein (*gfp*) fusion constructed downstream *opvB* (so that the strain remains OpvAB^+^). In the absence of phage, most *S*. *enterica* cells expressed *opvAB* at low levels; however, a small subpopulation that expressed *opvAB* at high levels was also detected. Phage challenge yielded mostly *S*. *enterica* cells with high levels of *opvAB* expression. These observations provide additional evidence that phages P22, 9NA, and Det7 kill the *opvAB* ^OFF^ subpopulation, and that *opvAB* ^ON^ cells overtake the culture.

### Reversibility of OpvAB-mediated bacteriophage resistance

**Fig 5:**
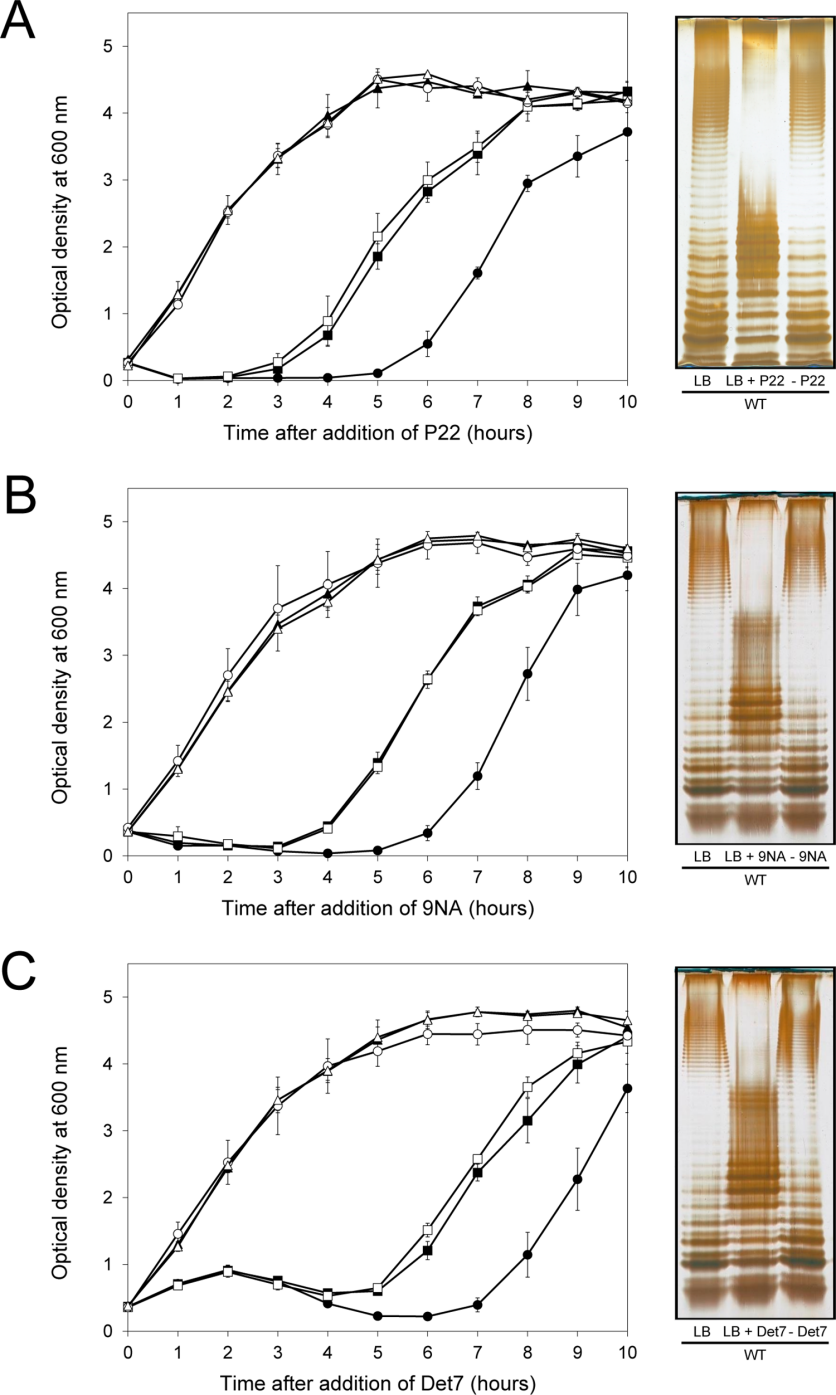
Reversibility of the phage-resistant phenotype in the wild type strain. **Left:** Growth of the wild type strain (blue squares), an *opvAB* ^ON^ strain (green triangles) and a Δ*opvAB* strain (red circles) in LB + P22 (A), LB + 9NA (B), and LB + Det7 (C). The same symbols in different colors (wild type in purple, *opvAB* ^ON^ in yellow, Δ*opvAB* in brown) indicate phage-resistant isolates which were re-challenged. Values are averages and standard deviations from ≥ 3 independent experiments. **Right:** LPS profiles of the wild type strain in LB (left), LB + phage (center) and of an isolate that had survived phage challenge, subsequently grown in LB (right). LPS profiles were obtained by electrophoresis and silver staining.

If the above model was correct, we reasoned, cessation of phage challenge should permit resuscitation of a phage-sensitive subpopulation as a consequence of *opvAB* phase variation. This prediction was tested by isolating single colonies from cultures in LB + phage. After removal of phage by streaking on green plates, individual isolates were cultured in LB and re-challenged with P22, 9NA, and Det7 (≥ 20 isolates for each phage). All were phage-sensitive and their LPS profile was identical to that obtained before phage challenge. Representative examples are shown in Fig 5. Unlike the wild type, individual isolates of the Δ*opvAB* strain remained phage-resistant after single colony isolation and were considered mutants (see below).

### Mutational bacteriophage resistance in the absence of OpvAB

**Fig 6:**
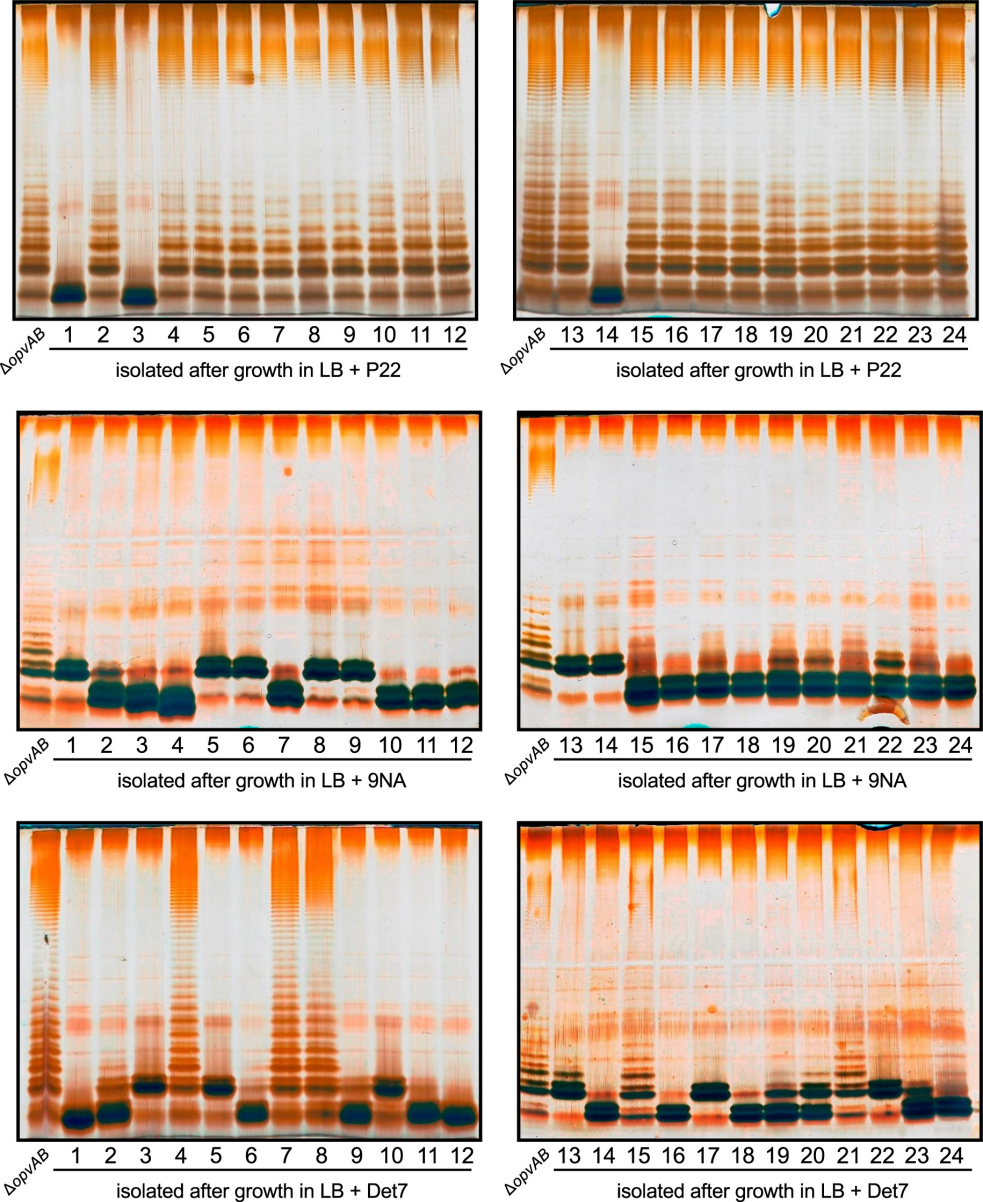
Mutational resistance to bacteriophage in a Δ*opvAB* strain. **Top.** LPS profiles of a *ΔopvAB* strain (first lane from the left on each gel) and 24 independent P22-resistant derivatives, as observed by electrophoresis and silver staining. **Middle.** LPS profiles of a *ΔopvAB* strain and 24 independent 9NA-resistant derivatives. **Bottom**. LPS profiles of a *ΔopvAB* strain and 24 independent Det7-resistant derivatives.

Challenge of a Δ*opvAB* strain with phages P22, 9NA, and Det7 prevented growth for 5–6 h, and growth resumed afterwards (Figs 3 and 5). To investigate the cause(s) of phage resistance in the absence of OpvAB, individual colonies were isolated from stationary cultures of a Δ*opvAB* strain in LB + P22, LB + 9NA, and LB + Det7. Phage was removed by streaking on green plates. Independent isolates (each from a different culture) were then tested for phage resistance. Sixty seven out of 72 independent isolates turned out to be phage-resistant, thus confirming that they were mutants. Analysis of LPS in independent phage-resistant mutants revealed that a large fraction of such mutants displayed visible LPS anomalies (Fig 6). The few mutant isolates (5/67) that did not show LPS alterations may have LPS alterations that cannot be detected in gels or carry mutations that confer phage resistance by mechanisms unrelated to the LPS. Whatever the case, these experiments support the conclusion that resistance of *S*. *enterica* to phages P22, 9NA, and Det7 in the absence of OpvAB is mutational.

To determine whether isolates resistant to one phage were also resistant to other phages that target the O-antigen, cross-resistance was tested by growth in LB upon phage inoculation. Sixty seven mutants (24 P22-resistant, 24 9NA-resistant, and 19 Det7-resistant) were tested (S1 Table). The main conclusions from these experiments were as follows:

1. Major alteration of LPS conferred resistance to all three phages.
2. More subtle LPS alteration (as observed in 16/24 P22-resistant mutants) conferred incomplete resistance to 9NA but did not confer resistance to Det7.

### Effect of *opvAB* expression on *Salmonella enterica* virulence

Because the LPS plays roles in the interaction between *S*. *enterica* and the animal host [34,46,47], we tested whether OpvAB-mediated alteration of O-antigen chain length affected *Salmonella* virulence. For this purpose, competitive indexes (CI's) [48] were calculated in the following experiments: (i) oral and intraperitoneal inoculation of BALB/c mice; (ii) infection of mouse macrophages *in vitro*; and (iii) exposure to guinea pig serum, an assay that provides reductionist assessment of the capacity of the pathogen to survive the bactericidal activity of complement [47]. As controls, CI's were also calculated in LB (Table 1). In all virulence assays, the CI of the *opvAB* ^ON^ strain was found to be lower than those of the wild type and the *ΔopvAB* strain. Because the wild type, the *opvAB* ^ON^ strain and the *ΔopvAB* strain show similar or identical growth rates in LB, the conclusion from these experiments was that expression of *opvAB* reduces *Salmonella* virulence (Table 1).

**Table 1:** Competitive indexes of *opvAB* ^ON^ and *ΔopvAB* strains of *S*. *enterica*.

Pair of strains	Competitive index^a^				
	LB broth	Mouse, oral infection	Mouse, intraperitoneal infection	Macrophages	Serum
*opvAB* ^ON^ *vs* wild type	1.03 ± 0.16	0.15 ± 0.07	0.32 ± 0.10	0.16 ± 0.09	0.14 ± 0.06
*ΔopvAB vs* wild type	1.14 ± 0.08	1.11 ± 0.18	1.15 ± 0.16	1.17 ± 0.24	1.04 ± 0.17
*opvAB* ^ON^ *vs ΔopvAB*	0.96 ± 0.13	0.25 ± 0.10	0.38 ± 0.15	0.23 ± 0.10	0.21 ± 0.04

^a^ Average and standard deviation from 4–10 experiments

## Discussion

A tradeoff is established whenever the adaptive capacity of an organism is increased at the expense of lowering the fitness conferred by specific phenotypic traits [49]. Tradeoffs have been mainly studied in sexually reproducing organisms but they occur also in microbes [50,51,52,53]. In pathogens, for instance, acquisition of mutational resistance to antimicrobial compounds often affects fitness [54,55], and may require loss of virulence as a payoff [56]. Bacteriophage resistance has been also shown to impair virulence in a variety of bacterial pathogens [57].

In this study, we describe a tradeoff that confers bacteriophage resistance at the expense of reducing virulence in the human pathogen *Salmonella enterica*. This tradeoff is however unusual because phage resistance is not mutational but epigenetic, and because the phage-resistant, avirulent phenotype is reversible.

The *opvAB* operon is present in most *Salmonella* serovars (S2 Table). Its products are inner membrane proteins that form intertwining ribbons (Fig 1) reminiscent of those formed by the LPS in the outer membrane [33]. Synthesis of OpvA and OpvB causes a decrease of long O-antigen chains and an increase of short O-antigen chains in the LPS (Fig 2; see also [32]). Genetic evidence presented in Fig 2 suggests that OpvA may prevent the formation of normal O-antigen, allowing OpvB to compete with the Wzz_ST_ modal length regulator. A similar phenomenon occurs in *Pseudomonas aeruginosa*, where the Iap transmembrane peptide encoded by bacteriophage D3 disrupts endogenous O-antigen biosynthesis allowing a phage-encoded O-antigen polymerase to produce a different O-antigen [58]. OpvB confers a predominant modal length of 3–8 units, while the wild type LPS shows modal lenghts of 16–35 units and of >100 units (Fig 2; see also [32]). As a consequence of the dramatic change in LPS structure caused by *opvAB* expression, *S*. *enterica* becomes resistant to bacteriophages 9NA, Det7, and P22 (Figs 3 and 4), an observation consistent with the fact that the O-antigen is the bacterial surface receptor used by these bacteriophages [39,59].

Expression of *opvAB* undergoes phase variation under the control of DNA adenine methylation and the transcriptional regulator OxyR [32]. Because *opvAB* phase variation is skewed towards the OFF state [32], *S*. *enterica* populations contain a major subpopulation of *opvAB* ^OFF^ (phage-sensitive) cells and a minor subpopulation of *opvAB* ^ON^ (phage-resistant) cells. In the presence of a bacteriophage that targets the O-antigen, the *opvAB* ^OFF^ subpopulation disappears and the *opvAB* ^ON^ subpopulation is selected (Figs 3 and 4). Hence, the existence of a small subpopulation of phage-resistant cells preadapts *S*. *enterica* to survive phage challenge. In OpvAB^−^ *S*. *enterica*, acquisition of phage resistance is mutational only, and a frequent mechanism is alteration of LPS structure (Fig 6). Because the LPS plays major roles in bacterial physiology including resistance to environmental injuries and host-pathogen interaction [60], *opvAB* phase variation may have selective value by providing *S*. *enterica* with a non-mutational, reversible mechanism of phage resistance. This mechanism offers the additional advantage of protecting *Salmonella* from multiple phages, perhaps from all phages that bind the O-antigen (note that the phages used in this study belong to three different families: Podoviridae, Siphoviridae, and Myoviridae).

Acquisition of phage resistance in *opvAB* ^ON^ cells requires a payoff: reduced virulence in both the mouse model and *in vitro* virulence assays (Table 1). In a phage-free environment, this payoff may be irrelevant because the avirulent subpopulation is minor as a consequence of skewed switching of *opvAB* toward the OFF state: 4 x 10^−2^ for ON→OFF switching *vs* 6 x 10^−5^ for OFF→ON switching [32]. In other words, only 1/1,000 *S*. *enterica* cells can be expected to be avirulent in a phage-free environment. The virulence payoff is therefore enforced in the presence of phage only, and its adaptive value may be obvious as it permits survival. On the other hand, the fitness cost of OpvAB-mediated phage resistance can be expected to be temporary because phase variation permits resuscitation of the virulent *opvAB* ^OFF^ subpopulation as soon as phage challenge ceases (Fig 5). Resuscitation may actually be rapid as a consequence of skewed switching towards the *opvAB* ^OFF^ state.

Phase variation systems that contribute to bacteriophage resistance have been described previously. For instance, certain restriction-modification systems show phase-variable expression [31]. However, protection by restriction-modification systems can be expected to be incomplete as only a fraction of infecting phage genomes are modified [61]. Phase variation can also confer phage resistance by preventing infection, and an interesting example is the *gtrABC1* cluster which protects *S*. *enterica* against the T5-like phage SPC35 [28]. Although the receptor of SPC35 is the BtuB vitamin transporter, GtrABC-mediated glycosylation of the LPS O-antigen may reduce SPC35 adsorption by an indirect mechanism [28]. In *Haemophilus influenzae*, phase-variable resistance to bacteriophage HP1c1 may involve changes in LPS [29]. Because these studies did not investigate the impact of phase variation on bacterial fitness, it remains unknown whether the tradeoff associated with *opvAB* phase variation is unusual or commonplace. However, if one considers that envelope structures play multiple roles in bacterial physiology aside from serving as phage receptors, it is tempting to predict that phase-variable bacteriophage resistance may frequently involve fitness costs. Whatever the payoff, however, phase-variable resistance may have a crucial advantage over mutation by creating phenotypic heterogeneity in a reversible manner.

## Methods

### Bacterial strains

Strains of *Salmonella enterica* used in this study (Table 2) belong to serovar Typhimurium, and derive from the mouse-virulent strain ATCC 14028. For simplicity, *S*. *enterica* serovar Typhimurium is routinely abbreviated as *S*. *enterica*. For the construction of strain SV7643, a fragment containing the promoterless *mCherry* gene and the kanamycin resistance cassette was PCR-amplified from pDOC-R, an mCherry-containing derivative of plasmid pDOC [62] using primers HindIII-opvB-mCherry-5 and NdeI-opvB-mCherry-3. The construct was integrated into the chromosome of *S*. *enterica* using the Lambda Red recombination system [63]. For the construction of strains SV5675, SV6786, SV6791, and SV8020, targeted gene disruption was achieved using plasmid pKD13 [63] and oligonucleotides listed in S3 Table: wzzB5-PS4 + wzzB3-PS1 for *wzz*_ST_ disruption, fepE5-PS4 + fepE3-PS1 for *wzz*_fepE_ disruption, STM2209-PS4tris + STM2209-PS1 for *opvA* disruption, and STM2208-PS4 + STM2208-PS1 for *opvB* disruption. The kanamycin resistance cassettes were then excised by recombination with plasmid pCP20 [63]. For the construction of strain SV6727, a fragment containing the promoterless green fluorescent protein (*gfp*) gene and the chloramphenicol resistance cassette was PCR-amplified from pZEP07 [64] using primers STM2208stop-GFP-5 and STM2208stop-GFP-3. The fragment was integrated into the chromosome of *S*. *enterica* using the Lambda Red recombination system [63]. An *opvB*::*gfp* transcriptional fusion was formed downstream of the *opvB* stop codon, and the strain remained OpvAB^+^. For the construction of strains SV7645, SV8117, and SV8118, plasmid pKD46 was introduced in SV6401, and the PCR products used for construction of strains SV7643, SV8020 and SV5675 were integrated into the chromosome of SV6401 using the Lambda Red recombination system [63].

**Table 2:** Strain list.

ATCC 14028	Wild type
SV5675	Δ*opvB*
SV6013^a^	Δ*opvAB*
SV6401^a^	*opvAB* ^ON^
SV6727	*opvB*::*gfp*
SV6786	Δ*wzz*_ST_
SV6791	Δ*wzz*_fepE_
SV6796	Δ*wzz*_ST_ Δ*wzz*_fepE_
SV7643	*opvB*::*mCherry*
SV7645	*opvB*::*mCherry* ^ON^
SV8020	Δ*opvA*
SV8117	Δ*opvA opvB* ^ON^
SV8118	*opvA* ^ON^ Δ*opvB*
SV8153	Δ*opvA opvB* ^ON^ *Δwzz*_ST_
SV8154	Δ*opvA opvB* ^ON^ Δ*wzz*_fepE_
SV8155	Δ*opvA opvB* ^ON^ Δ*wzz*_ST_ Δ*wzz*_fepE_

^a^ Strains described in [32].

### Culture media

Bertani's lysogeny broth (LB) was used as standard liquid medium. Solid LB contained agar at 1.5% final concentration. Green plates [65] contained methyl blue (Sigma-Aldrich, St. Louis, MO) instead of aniline blue. Antibiotics were used at the concentrations described previously [66].

### Bacteriophages

Bacteriophages 9NA [41,42] and Det7 [43] were kindly provided by Sherwood Casjens, University of Utah, Salt Lake City. Bacteriophage P22 H5 is a virulent derivative of bacteriophage P22 that carries a mutation in the *c2* gene [45], and was kindly provided by John R. Roth, University of California, Davis. For simplicity, P22 H5 is abbreviated as P22 throughout the text.

### Construction of plasmid pIZ2011

A DNA fragment containing *opvA* and the native *opvAB* promoter was PCR-amplified using primers KpnI-opvA-plasmidoGFP-5 and KpnI-opvA-plasmidoGFP-3 (S3 Table). The amplification product was cloned into pDOC-R [62]. The resulting plasmid produces an OpvA-mCherry fusion protein.

### Fluorescence microscopy

Bacterial cells from 1.5 ml of an exponential culture in LB at 37°C (OD_600_ ~0.15) were collected by centrifugation, washed in phosphate saline buffer (PBS), and resuspended in 1 ml of the same buffer. Cells were fixed in 4% formaldehyde solution and incubated at room temperature for 30 minutes. Finally, cells were washed, resuspended in PBS buffer, and stored at 4°C. Images were obtained by using an Olympus IX-70 Delta Vision fluorescence microscope equipped with a 100X UPLS Apo objective. Pictures were taken using a CoolSNAP HQ/ICX285 camera and analyzed using ImageJ software (Wayne Rasband, Research Services Branch, National Institute of Mental Health, MD, USA). Z-stacks (optical sections separated by 0.2 μm) of mCherry fluorescence were taken with the same microscope. Maximal intensity projections are shown.

### Electrophoretic visualization of LPS profiles

To investigate LPS profiles, bacterial cultures were grown in LB overnight. Bacterial cells were harvested and washed with 0.9% NaCl. The O.D._600_ of the washed bacterial suspension was measured to calculate cell concentration. A bacterial mass containing about 3 x 10^8^ cells was pelleted by centrifugation. Treatments applied to the bacterial pellet, electrophoresis of crude bacterial extracts, and silver staining procedures were performed as described by Buendia-Claveria *et al*. [67].

### Flow cytometry

Bacterial cultures were grown at 37°C in LB or LB + phage (P22, 9NA, or Det7) until exponential (OD_600_ ~0.3) or stationary phase (OD_600_ ~4). Cells were then diluted in PBS. Data acquisition and analysis were performed using a Cytomics FC500-MPL cytometer (Beckman Coulter, Brea, CA). Data were collected for 100,000 events per sample, and analyzed with CXP and FlowJo 8.7 software.

### Bacteriophage challenge

Overnight cultures were diluted 1:100 in 3 ml LB and grown in aeration by shaking at 37°C until they reached an optical density OD_600_ ~0.3. One hundred μl of a bacteriophage lysate (P22 H5, 9NA, or Det7) were added (M.O.I. ≥10), and OD_600_ was subsequently measured at 1 h intervals.

### Virulence assays

Eight-week-old female BALB/c mice (Charles River Laboratories, Santa Perpetua de Mogoda, Spain) were inoculated with pairwise combinations of the wild type, an *opvAB* ^ON^ strain, and a *ΔopvAB* strain at a 1:1 ratio. Bacterial cultures were previously grown overnight at 37°C in LB without shaking. Oral inoculation was performed by feeding the mice with 25 μl of PBS containing 0.1% lactose and 10^8^ bacterial colony-forming units (CFU). Intraperitoneal inoculation was performed with 10^4^ CFU in 200 μl of PBS. Bacteria were recovered from the spleen and the liver of infected mice at 2 days post-infection (intraperitoneal challenge) or 5 days post-infection (oral challenge). A competitive index (CI) was calculated as described elsewhere [48]. To permit strain discrimination, ATCC 14208 was tagged with *trg*::MudJ (Km^r^), an allele that is neutral for virulence [68]. When necessary, cross-streaking on green plates with P22 H5 was used to discriminate phage-resistant isolates [65]. Infection of cultured J774 mouse macrophages, inoculation of guinea pig serum (Sigma-Aldrich), and calculation of competitive indexes *in vitro* followed previously described protocols [68]. The Student's *t* test was used to determine whether the CI's were significant.

### Ethics statement

Animal research adhered to the principles mandatory in the European Union, as established in the Legislative Act 86/609 CEE (November 24, 1986) and followed the specific protocols established by the Royal Decree 1201/2005 of the Government of Spain (October 10, 2005). The protocols employed in the study were reviewed by the Comité Ético de Experimentación of the Consejo Superior de Investigaciones Científicas (CSIC), and were approved by the Consejería de Medio Ambiente, Comunidad de Madrid, Spain, on December 12, 2014 (permit number PROEX 257/14).

## Supporting Information

S1 Table Cross-resistance/sensitivity profiles of independent bacteriophage-resistant mutants obtained in a Δ*opvAB* background. (PDF)

S2 Table Distribution of the *opvAB* operon among *Salmonella enterica* serovars. (PDF)

S3 Table Oligonucleotides used in this study. (PDF)

S1 Fig Lipopolysaccharide structure. (PDF)

S2 Fig Growth curves of wild type, *opvAB* ^ON^ and *ΔopvAB* strains. (PDF)

## References

[1] Shapiro L (1976) Differentiation in the *Caulobacter* cell cycle. Annu Rev Microbiol 30: 377–407. 185940 10.1146/annurev.mi.30.100176.002113

[2] Kaiser D (1986) Control of multicellular development: *Dictyostelium* and *Myxococcus* . Annu Rev Genet 20: 539–566. 3028248 10.1146/annurev.ge.20.120186.002543

[3] Stragier P, Losick R (1996) Molecular genetics of sporulation in *Bacillus subtilis* . Annu Rev Genet 30: 297–241. 8982457 10.1146/annurev.genet.30.1.297

[4] Andrewes FW (1922) Studies in group agglutination. I. The *Salmonella* group and its antigenic structure. J Path Bact 25: 505–524.

[5] Legroux R, Magrou J (1920) État organisé des colonies bactériennes. Ann Inst Pasteur (Paris) 34: 417–431.

[6] Shapiro JA, Higgins NP (1989) Differential activity of a transposable element in *Escherichia coli* colonies. J Bacteriol 171: 5975–5986. 2553666 10.1128/jb.171.11.5975-5986.1989 PMC210462

[7] Shapiro JA (1998) Thinking about bacterial populations as multicellular organisms. Annu Rev Microbiol 52: 81–104. 9891794 10.1146/annurev.micro.52.1.81

[8] Zgur-Bertok D (2007) Phenotypic heterogeneity in bacterial populations. Acta Agric Slovenica 90: 17–24.

[9] Dhar N, McKinney JD (2007) Microbial phenotypic heterogeneity and antibiotic tolerance. Curr Opin Microbiol 10: 30–38. 17215163 10.1016/j.mib.2006.12.007

[10] Davidson CJ, Surette MG (2008) Individuality in bacteria. Annu Rev Genet 42: 253–268. 10.1146/annurev.genet.42.110807.091601 18652543

[11] Veening JW, Smits WK, Kuipers OP (2008) Bistability, epigenetics, and bet-hedging in bacteria. Annu Rev Microbiol 62: 193–210. 10.1146/annurev.micro.62.081307.163002 18537474

[12] Casadesus J, Low DA (2013) Programmed heterogeneity: epigenetic mechanisms in bacteria. J Biol Chem 288: 13929–13935. 10.1074/jbc.R113.472274 23592777 PMC3656251

[13] Thattai M, van Oudenaarden A (2004) Stochastic gene expression in fluctuating environments. Genetics 167: 523–530. 15166174 10.1534/genetics.167.1.523 PMC1470854

[14] Kussell E, Leibler S (2005) Phenotypic diversity, population growth, and information in fluctuating environments. Science 309: 2075–2078. 16123265 10.1126/science.1114383

[15] Wolf DM, Vazirani VV, Arkin AP (2005) Diversity in times of adversity: probabilistic strategies in microbial survival games. J Theor Biol 234: 227–253. 15757681 10.1016/j.jtbi.2004.11.020

[16] Hernandez SB, Cota I, Ducret A, Aussel L, Casadesus J (2012) Adaptation and preadaptation of *Salmonella enterica* to bile. PLoS Genet 8: e1002459 10.1371/journal.pgen.1002459 22275872 PMC3261920

[17] Sanchez-Romero MA, Casadesus J (2014) Contribution of phenotypic heterogeneity to adaptive antibiotic resistance. Proc Natl Acad Sci U S A 111: 355–360. 10.1073/pnas.1316084111 24351930 PMC3890857

[18] Ni M, Decrulle AL, Fontaine F, Demarez A, Taddei F, et al (2012) Pre-disposition and epigenetics govern variation in bacterial survival upon stress. PLoS Genet 8: e1003148 10.1371/journal.pgen.1003148 23284305 PMC3527273

[19] Silverman M, Zieg J, Hilmen M, Simon M (1979) Phase variation in *Salmonella*: genetic analysis of a recombinational switch. Proc Natl Acad Sci U S A 76: 391–395. 370828 10.1073/pnas.76.1.391 PMC382945

[20] Moxon ER, Rainey PB, Nowak MA, Lenski RE (1994) Adaptive evolution of highly mutable loci in pathogenic bacteria. Curr Biol 4: 24–33. 7922307 10.1016/s0960-9822(00)00005-1

[21] Moxon R, Bayliss C, Hood D (2006) Bacterial contingency loci: the role of simple sequence DNA repeats in bacterial adaptation. Annu Rev Genet 40: 307–333. 17094739 10.1146/annurev.genet.40.110405.090442

[22] Dubnau D, Losick R (2006) Bistability in bacteria. Mol Microbiol 61: 564–572. 16879639 10.1111/j.1365-2958.2006.05249.x

[23] Hernday A, Krabbe M, Braaten B, Low D (2002) Self-perpetuating epigenetic pili switches in bacteria. Proc Natl Acad Sci U S A 99: 16470–16476. 12202745 10.1073/pnas.182427199 PMC139910

[24] Casadesus J, Low D (2006) Epigenetic gene regulation in the bacterial world. Microbiol Mol Biol Rev 70: 830–856. 16959970 10.1128/MMBR.00016-06 PMC1594586

[25] van der Woude MW (2011) Phase variation: how to create and coordinate population diversity. Curr Opin Microbiol 14: 205–211. 10.1016/j.mib.2011.01.002 21292543

[26] van der Woude MW, Baumler AJ (2004) Phase and antigenic variation in bacteria. Clin Microbiol Rev 17: 581–611, table of contents. 15258095 10.1128/CMR.17.3.581-611.2004 PMC452554

[27] van der Woude MW (2006) Re-examining the role and random nature of phase variation. FEMS Microbiol Lett 254: 190–197. 16445745 10.1111/j.1574-6968.2005.00038.x

[28] Kim M, Ryu S (2012) Spontaneous and transient defence against bacteriophage by phase-variable glucosylation of O-antigen in *Salmonella enterica* serovar Typhimurium. Mol Microbiol 86: 411–425. 10.1111/j.1365-2958.2012.08202.x 22928771

[29] Zaleski P, Wojciechowski M, Piekarowicz A (2005) The role of Dam methylation in phase variation of *Haemophilus influenzae* genes involved in defence against phage infection. Microbiology 151: 3361–3369. 16207918 10.1099/mic.0.28184-0

[30] Srikhanta YN, Maguire TL, Stacey KJ, Grimmond SM, Jennings MP (2005) The phasevarion: a genetic system controlling coordinated, random switching of expression of multiple genes. Proc Natl Acad Sci U S A 102: 5547–5551. 15802471 10.1073/pnas.0501169102 PMC556257

[31] Hoskisson PA, Smith MC (2007) Hypervariation and phase variation in the bacteriophage 'resistome'. Curr Opin Microbiol 10: 396–400. 17719266 10.1016/j.mib.2007.04.003

[32] Cota I, Blanc-Potard AB, Casadesus J (2012) *STM2209-STM2208 (opvAB)*: a phase variation locus of *Salmonella enterica* involved in control of O-antigen chain length. PLoS One 7: e36863 10.1371/journal.pone.0036863 22606300 PMC3350482

[33] Ghosh AS, Young KD (2005) Helical disposition of proteins and lipopolysaccharide in the outer membrane of *Escherichia coli* . J Bacteriol 187: 1913–1922. 15743937 10.1128/JB.187.6.1913-1922.2005 PMC1064060

[34] Murray GL, Attridge SR, Morona R (2003) Regulation of *Salmonella typhimurium* lipopolysaccharide O antigen chain length is required for virulence; identification of FepE as a second Wzz. Mol Microbiol 47: 1395–1406. 12603743 10.1046/j.1365-2958.2003.03383.x

[35] Goldman RC, Hunt F (1990) Mechanism of O-antigen distribution in lipopolysaccharide. J Bacteriol 172: 5352–5359. 1697578 10.1128/jb.172.9.5352-5359.1990 PMC213199

[36] Bastin DA, Stevenson G, Brown PK, Haase A, Reeves PR (1993) Repeat unit polysaccharides of bacteria: a model for polymerization resembling that of ribosomes and fatty acid synthetase, with a novel mechanism for determining chain length. Mol Microbiol 7: 725–734. 7682279 10.1111/j.1365-2958.1993.tb01163.x

[37] Daniels C, Morona R (1999) Analysis of *Shigella flexneri* Wzz (Rol) function by mutagenesis and cross-linking: Wzz is able to oligomerize. Mol Microbiol 34: 181–194. 10540296 10.1046/j.1365-2958.1999.01591.x

[38] Batchelor RA, Alifano P, Biffali E, Hull SI, Hull RA (1992) Nucleotide sequences of the genes regulating O-polysaccharide antigen chain length (rol) from *Escherichia coli* and *Salmonella typhimurium*: protein homology and functional complementation. J Bacteriol 174: 5228–5236. 1379582 10.1128/jb.174.16.5228-5236.1992 PMC206356

[39] Lindberg AA (1973) Bacteriophage receptors. Annu Rev Microbiol 27: 205–241. 4584686 10.1146/annurev.mi.27.100173.001225

[40] Kintz E, Davies MR, Hammarlof DL, Canals R, Hinton JC, et al (2015) A BTP1 prophage gene present in invasive non-typhoidal *Salmonella* determines composition and length of the O-antigen of the lipopolysaccharide. Mol Microbiol 96: 263–275. 10.1111/mmi.12933 25586744 PMC4413052

[41] Wilkinson RG, Gemski P Jr., Stocker BA (1972) Non-smooth mutants of *Salmonella typhimurium*: differentiation by phage sensitivity and genetic mapping. J Gen Microbiol 70: 527–554. 4556257 10.1099/00221287-70-3-527

[42] Casjens SR, Leavitt JC, Hatfull GF, Hendrix RW (2014) Genome sequence of *Salmonella* phage 9NA. Genome Announc 2: e00531–14 10.1128/genomeA.00531-14 25146133 PMC4153490

[43] Walter M, Fiedler C, Grassl R, Biebl M, Rachel R, et al (2008) Structure of the receptor-binding protein of bacteriophage det7: a podoviral tail spike in a myovirus. J Virol 82: 2265–2273. 18077713 10.1128/JVI.01641-07 PMC2258939

[44] Casjens SR, Jacobs-Sera D, Hatfull GF, Hendrix RW (2015) Genome sequence of *Salmonella enterica* phage Det7. Genome Announc 3: e00279–00215. 10.1128/genomeA.00279-15 25953168 PMC4424284

[45] Smith HO, Levine M (1964) Two sequential repressions of DNA synthesis in the establishment of lysogeny by phage P22 and Its mutants. Proc Natl Acad Sci U S A 52: 356–363. 14206603 10.1073/pnas.52.2.356 PMC300284

[46] Raetz CR, Whitfield C (2002) Lipopolysaccharide endotoxins. Annu Rev Biochem 71: 635–700. 12045108 10.1146/annurev.biochem.71.110601.135414 PMC2569852

[47] Bravo D, Silva C, Carter JA, Hoare A, Alvarez SA, et al (2008) Growth-phase regulation of lipopolysaccharide O-antigen chain length influences serum resistance in serovars of *Salmonella* . J Med Microbiol 57: 938–946. 10.1099/jmm.0.47848-0 18628492

[48] Beuzon CR, Holden DW (2001) Use of mixed infections with *Salmonella* strains to study virulence genes and their interactions in vivo. Microbes Infect 3: 1345–1352. 11755424 10.1016/s1286-4579(01)01496-4

[49] Williams GC (1966) Natural selection, the costs of reproduction, and a refinement of Lack's principle. Am Nat 100: 687–690.

[50] Nystrom T (2004) Growth versus maintenance: a trade-off dictated by RNA polymerase availability and sigma factor competition? Mol Microbiol 54: 855–862. 15522072 10.1111/j.1365-2958.2004.04342.x

[51] Shoval O, Sheftel H, Shinar G, Hart Y, Ramote O, et al (2012) Evolutionary trade-offs, Pareto optimality, and the geometry of phenotype space. Science 336: 1157–1160. 10.1126/science.1217405 22539553

[52] Bailly-Bechet M, Benecke A, Hardt WD, Lanza V, Sturm A, et al (2011) An externally modulated, noise-driven switch for the regulation of SPI1 in *Salmonella enterica* serovar Typhimurium. J Math Biol 63: 637–662. 10.1007/s00285-010-0385-1 21107576

[53] Ferenci T, Spira B (2007) Variation in stress responses within a bacterial species and the indirect costs of stress resistance. Ann N Y Acad Sci 1113: 105–113. 17483210 10.1196/annals.1391.003

[54] Andersson DI, Hughes D (2010) Antibiotic resistance and its cost: is it possible to reverse resistance? Nat Rev Microbiol 8: 260–271. 10.1038/nrmicro2319 20208551

[55] De Paepe M, Gaboriau-Routhiau V, Rainteau D, Rakotobe S, Taddei F, et al (2011) Trade-off between bile resistance and nutritional competence drives *Escherichia coli* diversification in the mouse gut. PLoS Genet 7: e1002107 10.1371/journal.pgen.1002107 21698140 PMC3116916

[56] Vincent BM, Lancaster AK, Scherz-Shouval R, Whitesell L, Lindquist S (2013) Fitness trade-offs restrict the evolution of resistance to amphotericin B. PLoS Biol 11: e1001692 10.1371/journal.pbio.1001692 24204207 PMC3812114

[57] Leon M, Bastias R (2015) Virulence reduction in bacteriophage resistant bacteria. Front Microbiol 6: 343 10.3389/fmicb.2015.00343 25954266 PMC4407575

[58] Taylor VL, Udaskin ML, Islam ST, Lam JS (2013) The D3 bacteriophage alpha-polymerase inhibitor (Iap) peptide disrupts O-antigen biosynthesis through mimicry of the chain length regulator Wzz in *Pseudomonas aeruginosa* . J Bacteriol 195: 4735–4741. 10.1128/JB.00903-13 23955007 PMC3807437

[59] Susskind MM, Botstein D (1978) Molecular genetics of bacteriophage P22. Microbiol Rev 42: 385–413. 353481 10.1128/mr.42.2.385-413.1978 PMC281435

[60] Whitfield C, Trent MS (2014) Biosynthesis and export of bacterial lipopolysaccharides. Annu Rev Biochem 83: 99–128. 10.1146/annurev-biochem-060713-035600 24580642

[61] Wilson GG, Murray NE (1991) Restriction and modification systems. Annu Rev Genet 25: 585–627. 1812816 10.1146/annurev.ge.25.120191.003101

[62] Lee DJ, Bingle LE, Heurlier K, Pallen MJ, Penn CW, et al (2009) Gene doctoring: a method for recombineering in laboratory and pathogenic *Escherichia coli* strains. BMC Microbiol 9: 252 10.1186/1471-2180-9-252 20003185 PMC2796669

[63] Datsenko KA, Wanner BL (2000) One-step inactivation of chromosomal genes in *Escherichia coli* K-12 using PCR products. Proc Natl Acad Sci U S A 97: 6640–6645. 10829079 10.1073/pnas.120163297 PMC18686

[64] Hautefort I, Proenca MJ, Hinton JC (2003) Single-copy green fluorescent protein gene fusions allow accurate measurement of *Salmonella* gene expression in vitro and during infection of mammalian cells. Appl Environ Microbiol 69: 7480–7491. 14660401 10.1128/AEM.69.12.7480-7491.2003 PMC310007

[65] Chan RK, Botstein D, Watanabe T, Ogata Y (1972) Specialized transduction of tetracycline by phage P22 in *Salmonella typhimurium*. II. Properties of a high frequency transducing lysate. Virology 50: 883–898. 4565618 10.1016/0042-6822(72)90442-4

[66] Torreblanca J, Casadesús J (1996) DNA adenine methylase mutants of *Salmonella typhimurium* and a novel Dam-regulated locus. Genetics 144: 15–26. 8878670 10.1093/genetics/144.1.15 PMC1207489

[67] Buendia-Claveria AM, Moussaid A, Ollero FJ, Vinardell JM, Torres A, et al (2003) A purL mutant of *Sinorhizobium fredii* HH103 is symbiotically defective and altered in its lipopolysaccharide. Microbiology 149: 1807–1818. 12855732 10.1099/mic.0.26099-0

[68] Segura I, Casadesus J, Ramos-Morales F (2004) Use of mixed infections to study cell invasion and intracellular proliferation of *Salmonella enterica* in eukaryotic cell cultures. J Microbiol Methods 56: 83–91. 14706753 10.1016/j.mimet.2003.09.004

