# Supplementary material for: Epigenetic Control of Salmonella enterica O-Antigen Chain Length: A Tradeoff between Virulence and Bacteriophage Resistance
Source: PLoS Genet. 2015 Nov 19;11(11):e1005667. doi: 10.1371/journal.pgen.1005667 (PMC4652898; doi:10.1371/journal.pgen.1005667)
Supplement: S1 Table — (PDF) [file pgen.1005667.s001.pdf]

S1 Table. Cross-resistance/sensitivity profiles of independent bacteriophage-resistant mutants obtained in a Δ*opvAB* background. The designation of the isolate indicates the phage used to select the mutant.

	P22	9NA	Det7
Δ*opvAB*	S	S	S
opvAB^{ON}	R	R	R
P22-1	R	R	R
P22-2	R	R/S	S
P22-3	R	R	R
P22-4	R	R/S	S
P22-5	R	R/S	S
P22-6	R	R/S	S
P22-7	R	R/S	S
P22-8	R	R/S	S
P22-9	R	R/S	S
P22-10	R	R/S	S
P22-11	R	R/S	S
P22-12	R	R/S	S
P22-13	R	R/S	S
P22-14	R	R	R
P22-15	R	R/S	S
P22-16	R	R/S	S
P22-17	R	R/S	S
P22-18	R	R/S	S
P22-19	R	R/S	S
P22-20	R	R/S	S
P22-21	R	R/S	S
P22-22	R	R/S	S
P22-23	R	R/S	S
P22-24	R	R/S	S
9NA-1	R	R	R
9NA-2	R	R	R
9NA-3	R	R	R
9NA-4	R	R	R
9NA-5	R	R	R
9NA-6	R	R	R
9NA-7	R	R	R
9NA-8	R	R	R
9NA-9	R	R	R
9NA-10	R	R	R
9NA-11	R	R	R
9NA-12	R	R	R
9NA-13	R	R	R
9NA-14	R	R	R
9NA-15	R	R	R
9NA-16	R	R	R
9NA-17	R	R	R
9NA-18	R	R	R
9NA-19	R	R	R
9NA-20	R	R	R

9NA-21	R	R	R
9NA-22	R	R	R
9NA-23	R	R	R
9NA-24	R	R	R
Det7-1	R	R	R
Det7-2	R	R	R
Det7-3	R	R	R
Det7-5	R	R	R
Det7-6	R	R	R
Det7-9	R	R	R
Det7-10	R	R	R
Det7-11	R	R	R
Det7-12	R	R	R
Det7-13	R	R	R
Det7-14	R	R	R
Det7-16	R	R	R
Det7-17	R	R	R
Det7-18	R	R	R
Det7-19	R	R	R
Det7-20	R	R	R
Det7-22	R	R	R
Det7-23	R	R	R
Det7-24	R	R	R

R: reSiStant. S: SenSitive. R/S: intermediate.
