# Supplementary material for: Epigenetic Control of Salmonella enterica O-Antigen Chain Length: A Tradeoff between Virulence and Bacteriophage Resistance
Source: PLoS Genet. 2015 Nov 19;11(11):e1005667. doi: 10.1371/journal.pgen.1005667 (PMC4652898; doi:10.1371/journal.pgen.1005667)
Supplement: S2 Table — (PDF) [file pgen.1005667.s002.pdf]

S2 Table. Distribution of the *opvAB* operon among *Salmonella enterica* serovars

**Subspecies**	**Serovar**	**Strain**	***opvAB***	**GenBank accession number**
enterica (I)	Agona	SL483	+	CP001138.1
	Anatum	ATCC BAA-1592	+	CP007531.1
	Bareilly	CFSAN000189	+	CP006053.1
	Bovismorbificans	3114	+	HF969015.2
	Choleraesuis	ATCC 10708	+	CP012344.1
	Dublin	CT_02021853	+	CP001144.1
	Enteritidis	SEE1	+	CP011790.1
	Gallinarum	287/91	+	AM933173.1
	Heidelberg	SL476	+	CP001120.1
	Infantis	Not specified	+	LN649235.1
	Montevideo	507440-20	+	CP007530.1
	Newport	SL254	+	CP001113.1
	Paratyphi A	CMCC50093	+	CP011967.1
	Typhi	Ty2	+	AE014613.1
	Typhimurium	ATCC 14028	+	CP001363.1
		LT2	+	AE006468.1
		SL1344	+	FQ312003.1
salamae (II)	Not specified	DMA-1	+	ATFA01000011.1
	58:l,z13,z28:z6	00-0163	+	AOXE01000063.1
	Not specified	RKS2993	+	JXTT01000070.1
	Not specified	3588/07	+	CAFD01000110.1
arizonae (IIIa)	62:z4,z23:--	Not specified	+	CP000880.1
	62:z36:-	RKS2983	+	CP006693.1
diarizonae (IIIb)	60:r:e,n,x,z15	01-0170	+	APAC01000077.1
houtenae (IV)	Not specified	ATCC BAA-1581	-	CM001471.1
indica (VI)	6,14,25:z10:1,(2),7	1121	+	AOXI01000025.1
