# Supplementary material for: Epigenetic Control of Salmonella enterica O-Antigen Chain Length: A Tradeoff between Virulence and Bacteriophage Resistance
Source: PLoS Genet. 2015 Nov 19;11(11):e1005667. doi: 10.1371/journal.pgen.1005667 (PMC4652898; doi:10.1371/journal.pgen.1005667)
Supplement: S3 Table — (PDF) [file pgen.1005667.s003.pdf]

S3 Table. Oligonucleotides used in this study

Name	Sequence (5'→3')
fepE3-PS1	ATCGCCAGCGCGTTTTCCATTTTACGCGAGACCATCG CGTGTGTAGGCTGGAGCTGCTTC
fepE5-PS4	ACAAGAAAAAAATCAGTCATTTGCAGGTTATTCACTGC CGATTCCGGGGATCCGTCGACC
fepE-E1	AAACTATCGGGCCCATCATC
fepE-E2	TCCTGCATGACCTGAATCAG
HindIII-opvB-mCherry-5	GCTAAGCTTAGAATATCGTATTGAGAAAAAGACAATGA ATGACCGCGCAATGGTGAGCAAGGGCGAGGA
KpnI-opvA-plasmidoGFP-3	TTTTGGTACCCATCCCTTTTAAAACGCTAA
KpnI-opvA-plasmidoGFP-5	TTTTGGTACCCTGATCATGATGACGTCCAC
mCherry	TGATGGCCATGTTATCCTCC
NdeI-opvB-mCherry-3	CGACATATGTTTGACACATTTCAGTGCAGAGTTTATCT CTGCGCAATGTAGTCACGACGTTGTAAAACG
STM2208-E1	AATATACGTTCGCAGCCAGG
STM2208-E2	TTCGACACATTTCAGCGCAG
STM2208-PS1	GCGCGGTCATTCATTGTCTTTTTCTCAATACGATATTC GGTGTAGGCTGGAGCTGCTTC
STM2208-PS4	GGGAGAAAATAGTGAAATTGATTTAGTCGACGTTTCCT TAATTCCGGGGATCCGTCGACC
STM2208stop-GFP-3	ACTTTTACTCTTCGACACATTTCAGCGCAGAGTTTATC CTGCGCAATGTTTATCACTTATTCAGGCGTA
STM2208stop-GFP-5	CGCTAACAGAATATCGTATTGAGAAAAAGACAATGAAT GACCGCGCATGATAAGAAGGAGATATACATATGAG
STM2209-E1	TTACCGATCGATATAACCAG
STM2209-E2	TTGTATCATGCTGCACGCTC

STM2209-PS1	TTTCACTATTTTCTCCCCGCATTTCACATCCCTTTTAAA AGTGTAGGCTGGAGCTGCTTC
STM2209-PS4tris	AATTCTTATGTGTGGGTTTTATCTTATGAAGAAATATAC GATTCCGGGGATCCGTCGACC
wzzB3-PS1	TAGCTACGTAGCGCATTGCGTCCCAGCACAATCCCGG CACGTGTAGGCTGGAGCTGCTTC
wzzB5-PS4	GTCTTCCGGGCGTGGGAACGATCCGGAACAGATTGAT TTGATTCCGGGGATCCGTCGACC
wzzB-E1	AGAGTGGCTCCGATAACTTC
wzzB-E2	ATCAACTGGAGCAGCTACTG
