# Supplementary material for: Epigenetic Control of Salmonella enterica O-Antigen Chain Length: A Tradeoff between Virulence and Bacteriophage Resistance
Source: PLoS Genet. 2015 Nov 19;11(11):e1005667. doi: 10.1371/journal.pgen.1005667 (PMC4652898; doi:10.1371/journal.pgen.1005667)
Supplement: S1 Fig — (PDF) [file pgen.1005667.s004.pdf]

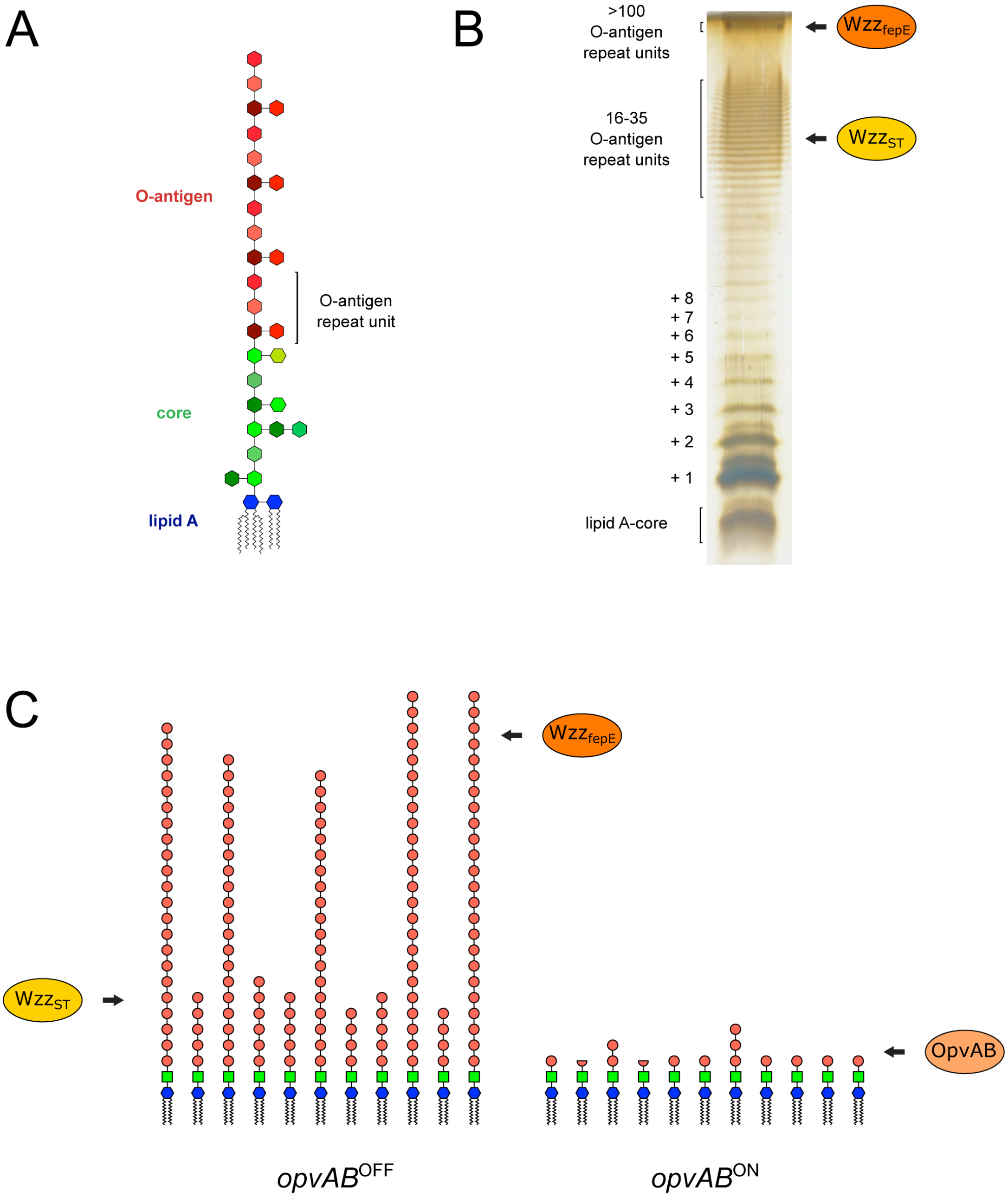

S1 Figure. A. Diagram of lipopolysaccharide structure. For simplicity, only four O-antigen repeat units are represented, but individual lipopolysaccharide molecules can have more than 100 O-antigen repeat units. **B.** Typical lipopolysaccharide profile of *Salmonella enterica* serovar Typhimurium strain ATCC 14028. Modal lengths conferred by Wzz_{ST} (16-35 O-antigen repeat units) and Wzz_{fepE} (>100 O-antigen repeat units) are indicated. **C.** Diagrams of lipopolysaccharide structure in *opvAB*^{OFF} and *opvAB*^{ON} subpopulations. Every red circle represents five O-antigen repeat units. Modal lengths conferred by Wzz_{ST}, Wzz_{fepE}, and OpvAB are indicated.
