# Supplementary material for: Epigenetic Control of Salmonella enterica O-Antigen Chain Length: A Tradeoff between Virulence and Bacteriophage Resistance
Source: PLoS Genet. 2015 Nov 19;11(11):e1005667. doi: 10.1371/journal.pgen.1005667 (PMC4652898; doi:10.1371/journal.pgen.1005667)
Supplement: S2 Fig — (PDF) [file pgen.1005667.s005.pdf]

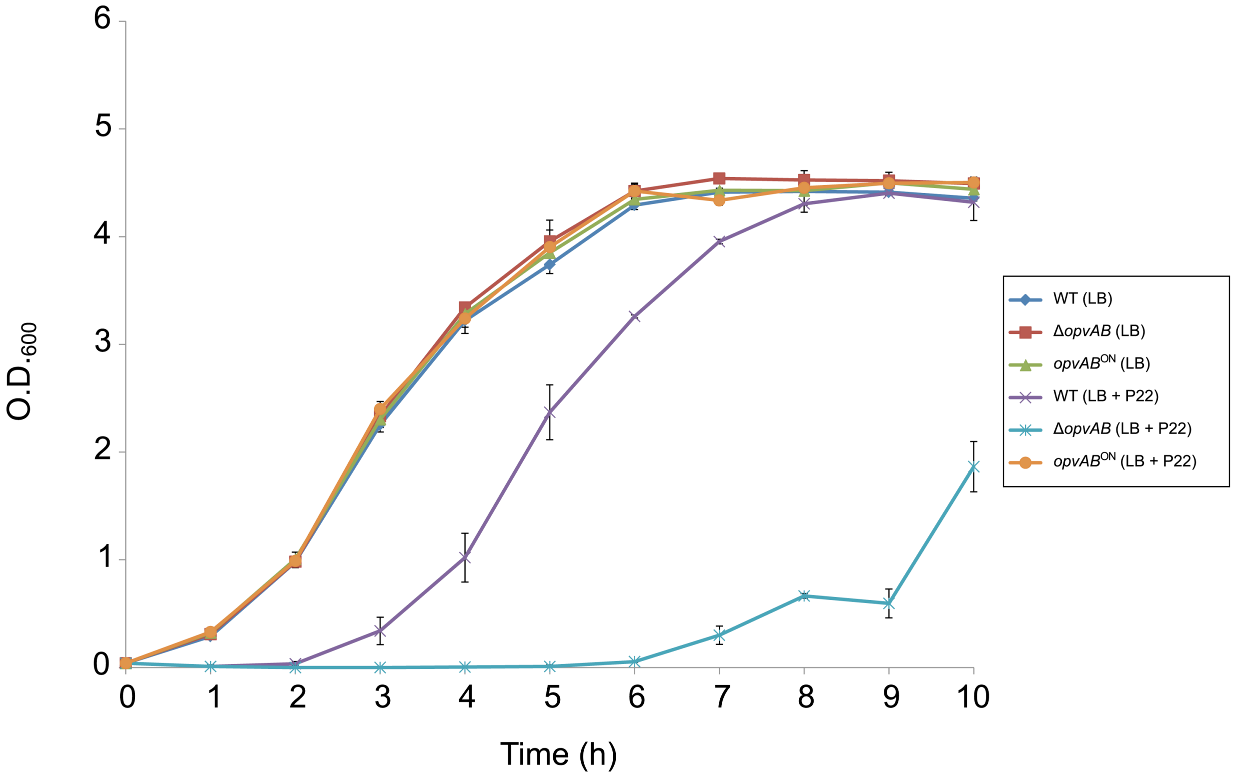

S2 Figure. Growth curves of wild type (ATCC 14028), *ΔopvAB*, and *opvAB*^{ON} strains in LB and in LB + P22 H5, plotted from average values and standard deviations from >4 independent experiments.
